# An outbreak of *Salmonella* Infantis linked to shredded pork products from an unlicensed source in multiple health districts, Ontario, Canada, 2021

**DOI:** 10.14745/ccdr.v50i05a06

**Published:** 2024-05-24

**Authors:** Victoria Osasah, Yvonne Whitfield, Affan Danish, Allana Murphy, Richard Mather, Janica Adams, Anna Majury, Mehdi Aloosh

**Affiliations:** 1 Enteric, Zoonotic and Vector-Borne Diseases, Public Health Ontario, Toronto, ON; 2 Public Health Ontario Laboratory, Public Health Ontario, Toronto, ON; 3 Department of Health Research Methods, Evidence, and Impact, Michael G. DeGroote School of Medicine, McMaster University, Hamilton, ON

**Keywords:** *Salmonella*, Infantis, epidemiologic studies, outbreak, foodborne, unlicensed source, restaurant

## Abstract

**Background:**

An outbreak of *Salmonella* Infantis was associated with the consumption of shredded pork products at multiple restaurants in Ontario between July 2021 and October 2021. The outbreak involved 36 case-patients from six public health units. The implicated shredded pork products were obtained from an unlicensed source. This is the largest reported outbreak of *Salmonella* Infantis linked to restaurant food exposures in Ontario, with complexities related to the investigation of unlicensed foods. This article aims to describe the epidemiological, food safety and laboratory investigations that led to the identification and removal of the source of the outbreak from implicated restaurants, including the challenges encountered while investigating an outbreak related to an unlicensed source of food.

**Methods:**

Epidemiological and laboratory analyses were conducted to identify the source of the outbreak. Food safety investigations were conducted to ascertain the origin and distribution of the implicated food.

**Results:**

Whole-genome sequencing identified the outbreak strain from the isolates of 36 case-patients across six public health units in Ontario. Seven case-patients (19%) were hospitalized. No deaths were reported. The outbreak was linked to shredded pork products (i.e., rinds or skins) that were distributed by an unlicensed meat processor and consumed at various restaurants that served Southeast Asian fusion cuisine concentrated in the Greater Toronto Area. The product was removed from implicated restaurants.

**Conclusion:**

Historically, foods from unlicensed sources have been implicated in multiple large outbreaks and continue to be of significant public health risk. The outbreak investigation emphasized the threat of food from unlicensed sources to the public’s health and the importance of additional public health interventions to prevent outbreaks linked to unlicensed sources.

## Introduction

### Identification

In August 2021, Public Health Ontario (PHO) identified, via routine surveillance, nine cases of *Salmonella* Infantis infection within 0–7 allele differences by whole-genome multi-locus sequence typing (wgMLST). Further follow up with local public health units identified a cluster of five case-patients who dined at a common restaurant serving Southeast Asian fusion cuisine. Four additional cases reporting similar exposures were identified in three additional jurisdictions within Ontario. A total of 17 Southeast Asian fusion restaurants were implicated. This led to the activation of the Ontario Outbreak Investigation Coordinating Committee on September 10, 2021. This committee is composed of local, provincial and federal partners who jointly convened and undertook the outbreak investigation.

### Background

In Ontario, salmonellosis is the second most common cause ((1)) of notifiable gastrointestinal infection ((2)). Over the past five years, approximately two per 1,000 persons per year in Ontario have experienced an illness from salmonellosis. An average of three per 100 persons are hospitalized annually in Ontario ((2)). Among the ten most reported serovars in the province, *Salmonella* Infantis is the fourth leading serovar ((3)).

The most notable outbreak of *Salmonella* Infantis in Ontario was reported in 1999 and linked to pig ear treats for pets ((4)). Nationally and internationally, outbreaks of *Salmonella* Infantis have been linked to poultry products as the vehicle of infection ((5,6)). An international outbreak of *Salmonella* Infantis linked to pork products was reported in Germany ((7)). Overall, the contamination of pork with *Salmonella* Infantis has been well described in the literature ((6,8)). According to FoodNet Canada, data have frequently identified *Salmonella* Infantis within poultry and pork products ((9)).

In the past decade, multiple outbreaks of salmonellosis have been associated with the distribution of food from unlicensed sources across Canada and the United States (US). Two of the larger outbreaks were linked to the distribution of food from commercial vendors (e.g., mobile food trucks and a catering company) using food from unlicensed sources ((10–12)). These outbreaks caused by foods from unlicensed sources have historically contributed to delays in the identification of the source of the infection, partly due to the distributor’s deviations from the standard regulatory practices necessary to track and stop the distribution and use of the implicated food products. These delays have significantly impeded timely public health investigations to identify the source and prevent further distribution. A secondary consequence is the sustained availability of the implicated food for consumption while investigators are conducting investigations to identify the source, potentially contributing to an increased incidence of cases with the disease-causing organism.

### Objective

Given the magnitude of this outbreak as observed with the large number of reported illnesses and its occurrence in restaurants across a wide geographical area, it was imperative to understand the epidemiology of the outbreak and the implications of distributing unlicensed food sources on the food investigation into the source of the outbreak. This article describes the epidemiological, laboratory and food safety investigations and the food safety challenges encountered and actions taken during the outbreak while investigating food from an unlicensed source.

## Methods

### Overview

Following an increase above the average count of case-patients that were linked by whole-genome sequencing (WGS), an outbreak was declared on September 10, 2021. By late October 2021, no additional cases were reported. The outbreak was declared over on November 11, 2021, following the identification and the removal of the source of the outbreak and a return below the average count of cases.

### Case finding and data collection

The Ontario Outbreak Investigation Coordinating Committee defined an outbreak confirmed case as an infection with *Salmonella* Infantis occurring among residents or visitors in Ontario, with a genomic sequence pattern (0–7 wgMLST allele differences) consistent with the outbreak strain, and an illness onset on or after July 12, 2021. Routine data for *Salmonella* are provided by PHO’s laboratory. On average, PHO’s laboratory reports two *Salmonella* Infantis cases per week in Ontario.

We conducted a descriptive study using standardized hypothesis-generating questionnaires in conjunction with laboratory data on clinical and food isolates. Ethics approval was not required as this study fell within the purview of PHO’s legislated mandate ((13)).

Case-patients with laboratory-confirmed *Salmonella* infections related to the outbreak strain were interviewed by local public health investigators, using a standardized hypothesis-generating questionnaire to obtain food, animal, water and occupational exposures during the seven-day period prior to the onset of the illness. Re-interviews of case-patients were also conducted by provincial investigators who collected additional exposure information to identify the source of the outbreak. Based on the information from the initial interviews, investigators inquired about exposure to pork and pork products, and obtained information on the location of the food purchase and consumption, including the name of the dish consumed. On September 13, 2021, a public health alert was issued on the Canadian Network for Public Health Intelligence to communicate the situation to public health partners.

## Investigations

### Laboratory investigations

In response to the outbreak investigation, clinical specimens obtained from case-patients who were part of the outbreak, and food specimens, including intact and opened specimens, obtained from locations where case-patients reported consuming food prior to illness, were analyzed at PHO’s laboratory and the Public Health Agency of Canada’s National Microbiology Laboratory. Public Health Ontario’s laboratory carried out real time polymerase chain reaction (PCR) analysis using the AOAC Research Institute 031001 method for food samples submitted by public health units for *Salmonella* detection ((14)). All positive and indeterminate real-time PCR analysis samples were transitioned to the Health Canada reference selective method (MFHPB-20) ((15)) for culture-based identification, and with serotyping confirmation via traditional phenotypic agglutination ((16)). Cluster analysis was routinely performed on all positive samples using the PulseNet Canada wgMLST approach, which defines screening for genomic relatedness as ≤10 wgMLST allele differences between samples ((17)). Presumptive isolates from the reference culture method were confirmed by the enteric laboratory at PHO. Specimens with culture-positive isolates were sent for confirmatory WGS to the National Microbiology Laboratory. Isolates within 0–7 wgMLST were identified as closely related to each other.

### Epidemiologic investigation

A binomial probability test was applied for the comparison of the proportions of the food exposures reported by the case-patients and the reference values from Foodbook survey respondents. The Foodbook Report is a population-based telephone survey conducted in all Canadian provinces within a one-year study period between 2014 and 2015 on food and animal exposure within a seven-day recall period ((18)). Microsoft Excel was used to conduct data analysis and to create epidemiological graphs. A significance level of *p*=0.05 was used.

Food reported by case-patients with higher than expected proportions to the reference values and with statistical significance (*p*<0.05) were further explored to identify similarities by purchase location, type of food and food ingredients. Information on restaurant exposures from the standardized hypothesis-generating questionnaires reported by case-patients was further analyzed to identify case-patients that dined at the same Southeast Asian fusion restaurants. A sub-analysis of the food exposures from case-patients that dined at the same restaurants was conducted and compared with other case-patients involved in the outbreak to identify a common source. In addition, household clustering was explored to identify case-patients who may have been exposed via non-primary transmission.

### Food safety investigation

Local, provincial and federal food safety investigators conducted site visits at the restaurants where case-patients reported dining and investigated the implicated food product. Food items that case-patients reported consuming and were suspected as the source of illness based on epidemiological data were obtained from the restaurants, including specimens from an affiliated restaurant serving similar meals without any reported illnesses.

## Results

### Epidemiologic findings

Thirty-six case patients that met the confirmed case definition were reported across six public health units. Majority of case patients (97%) were reported across five public health units within the Greater Toronto Area (GTA) between July and October 2021. No cases were identified outside of Ontario. The median age was 26 years (range: 0–94 years). Among all case patients, clustering was observed by gender as twenty-four (67%) case patients were male. Fourteen case patients (39%) were between 10 and 29 years. Seven case patients (19%) were hospitalized during the outbreak. No deaths were reported.

**Figure 1:**
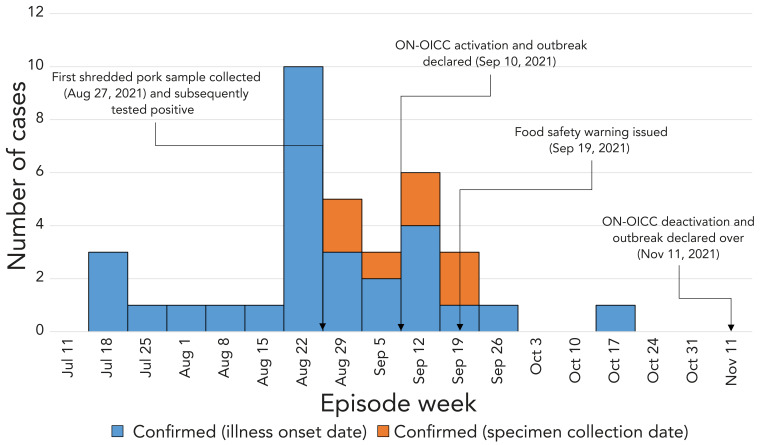
An epidemic curve of case-patients with *Salmonella* Infantis infections reported by week of illness onset or specimen collection date Ontario, July 18, 2021–October 17, 2021, (n=36) Abbreviation: ON-OICC, Ontario Outbreak Investigation Coordinating Committee

Illness onset dates ranged from July 19, 2021, to October 17, 2021, with multiple temporal clusters, within the three-month span, more than one incubation period apart, and with several peaks and valleys throughout the outbreak. These clusters reflect the dining pattern of the case patients and the fact that the outbreak was restaurant-based and involved a frozen contaminated food item with a long shelf life (**Figure 1**, **Table 1**).

**Table 1:** A distribution of the outbreak confirmed cases involved in the outbreak by illness onset date and specimen collection date, Ontario, July–October 2021

Episode week (2021)	Confirmed (illness onset date)	Confirmed (specimen collection date)
Jul 11	0	0
Jul 18	3	0
Jul 25	1	0
Aug 1	1	0
Aug 8	1	0
Aug 15	1	0
Aug 22	10	0
Aug 29	3	2
Sep 5	2	1
Sep 12	4	2
Sep 19	1	2
Sep 26	1	0
Oct 3	0	0
Oct 10	0	0
Oct 17	1	0
Oct 24	0	0
Oct 31	0	0
Nov 11	0	0

Exposure information was collected from a total of 30 case-patients (response rate: 83%) as five case-patients were lost to follow-up and one case-patient was unwilling to be interviewed. Among the 24 case-patients that provided a response for “consumption of pork”, 23 case-patients (96%) responded that they either consumed or probably consumed pork, representing a higher than expected proportion than the average proportion of the general population surveyed in the Foodbook Report (61%, *p*<0.005). Among the 23 case-patients that reported consuming or probably consuming pork, 19 (83%) case-patients reported consuming shredded pork rind, pork skin or a combination with pork chops at 17 restaurants serving Southeast Asian fusion cuisine in the GTA.

Additional food exposures were explored to determine if other food exposures could have been the source of the outbreak; however, the food items reported were identified as part of the dish served with shredded pork products and lacked differences among case-patients. Additionally, laboratory evidence strengthened the hypothesis that these food items were not the source of the outbreak. Of the 23 case-patients reporting consumption of pork, a total of 20 case-patients (87%) reported dining at restaurants and consuming pork. Of the 17 restaurant locations, there were three chains involved with clustering of three or more case-patients per restaurant chain. These accounted for 12 case-patients. The remaining eight cases dined at eight different individual locations.

### Food safety investigations

**Figure 2:**
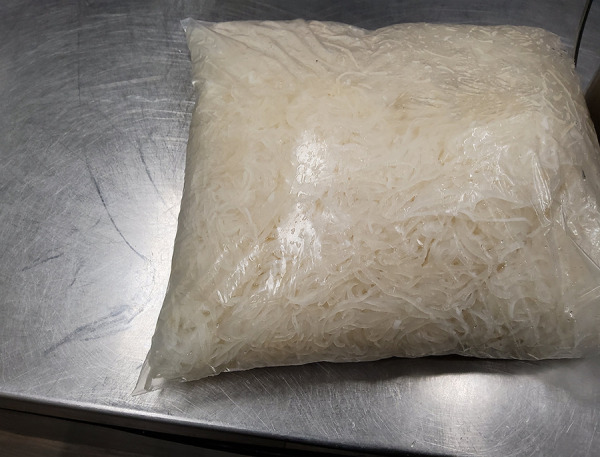
A picture of the implicated shredded pork product taken by investigators and issued in a Food Safety Warning^a^ ^a^ Information can be found on the Canadian Food Inspection Agency’s Food Safety Warning page

Investigations to trace the distribution of the implicated product included the collection of shredded pork products from the restaurants. It was determined that the shredded pork products were sold frozen in transparent plastic bags, with no labels, no lot codes, no identifiers and no cooking instructions. Pictures obtained of the unlabelled products (**Figure 2**) aided investigators in identifying and removing similar products from use from all restaurants serving Southeast Asian fusion cuisines across the GTA. The pictures also aided in identifying additional shredded pork samples for testing.

There were no cooking instructions on the package to determine if it was a ready-to-eat product or if additional cooking was required. Some restaurant operators reported that they served the shredded pork products without additional heat treatment.

Further investigations revealed that all the restaurants shared a common meat processor of the specific shredded pork products. At the time of the outbreak investigation, the meat processor operated without a license. One of the challenges during the trace back investigation involved obtaining contact information of the meat processor from the restaurant operators. Some restaurant operators could only provide the name and telephone number of the meat processor. The contact information provided by restaurants were the same; however, investigators were unable to establish contact with the meat processor. Therefore, further information could not be obtained.

Another challenge that occurred at the restaurant-level was obtaining accurate information about the source of the shredded pork products that were purchased. Upon re-inspection, some restaurant operators provided conflicting information about the purchase source of the shredded pork products. The operators implicated a licensed supplier after initially identifying the unlicensed supplier as the purchase source. The licensed supplier was inspected and food specimens were collected. *Salmonella* was not detected in any of the food specimens that were obtained from the licensed supplier.

### Laboratory findings

An initial laboratory analysis identified that a *Salmonella* Infantis isolate from an intact shredded pork product sample, obtained from one of the implicated restaurants was related by WGS to the outbreak strain. In total, 75 food samples, including 37 samples of shredded pork rind, shredded pork skin and mixed pork chops, were obtained from 17 implicated restaurants, one restaurant with no known case-patients reported, all 18 restaurants serving Southeast Asian fusion cuisine, and one private residence during the investigation. The other 38 food samples tested included rice (n=5), chicken (n=5), vegetables (n=4), beef (n=4), egg (n=3), sausage (n=2), spring rolls (n=2), tofu (n=2), vermicelli (n=2), salsa (n=2) and one sample each of sour cream, tortilla, peppers, cheese, duck, teriyaki sandwich and rice powder.

**Figure 3:**
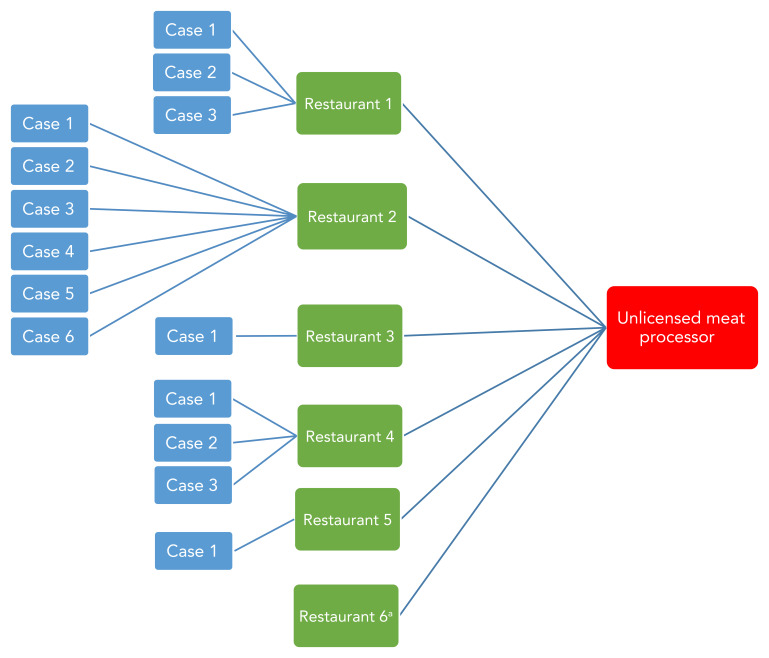
A trace back diagram of the restaurants and number of case-patients linked to each restaurant from which the 14 positive shredded pork product isolates were obtained, Ontario, July–October 2021^a^ ^a^ No cases were reported linked to Restaurant 6

Fourteen positive food isolates from the shredded pork products obtained from seven restaurants (39%) (**Figure 3**) shared similar genetic patterns with the outbreak strain by WGS. The isolates were within seven allele differences by wgMLST from each other. Isolates from clinical specimens obtained from the case-patients were also within seven allele range by wgMLST.

### Public health interventions

Provincial and federal investigators issued a food safety warning ((19)) (Figure 2) to the public as well as to hotels, restaurants and institutions against the use of this shredded pork product. In addition, once the implicated product was identified, local investigators conducted inspections of all Southeast Asian fusion style restaurants within their jurisdictions. Investigators provided education to restaurant operators and removed the implicated shredded pork products wherever found. No additional restaurants were identified.

## Discussion

### Key results

Shredded pork products were identified as the source of the outbreak that led to 36 instances of illness among 17 restaurants across five health districts in the GTA and one health district outside the GTA. It was hypothesized that contamination at various stages of the food processing continuum could have occurred in the supply chain, providing multiple opportunities for the contamination and transmission of *Salmonella* Infantis in the pork products. Furthermore, undercooking and inadequate processing of the shredded pork products at either the processor or restaurant level may have occurred. The shredded pork products were unlabelled without any cooking instructions and were not further cooked at the restaurant level. The large-scale distribution of these unlicensed pork products to numerous restaurants was an unusual occurrence in Ontario.

### Comparison to other *Salmonella* Infantis outbreaks and outbreaks from unlicensed sources

Although *Salmonella* Infantis is a common serovar in Ontario and has been associated with multiple outbreaks ((8,20)), this outbreak presented some unusual investigation patterns that were different from previous outbreak events of *Salmonella* Infantis in Ontario, mainly due to widespread distribution of unlicensed pork products to restaurants. Previous foodborne outbreaks of *Salmonella* Infantis in Canada have been associated with food sold at retail stores and consumed at home ((4,20,21)). This was the largest reported restaurant associated outbreak of *Salmonella* Infantis in Ontario, resulting from the consumption of food from an unlicensed food processor. However, the magnitude of the outbreak, tight clustering of cases both spatially and temporally, and distribution to large-scale gatherings (such as restaurants) was consistent with previous outbreaks associated with food from unlicensed sources ((10–12)). Previous outbreaks involving unlicensed sources have exemplified the impact of food from unlicensed sources on investigations into the identification of the source of an outbreak. This outbreak faced additional challenges not reported in the existing literature, which included limited available information on the distributor and manufacturer of the product and often conflicting information from restaurant operators who provided inconsistent information about the vendor’s name and location. This posed a challenge for investigators in identifying the source of the outbreak in a timely manner. Some operators were fined for obtaining food from unlicensed sources.

## Limitations

There were several limitations involved with obtaining information from case-patients. Since information was obtained from case-patients following the outbreak, we cannot rule out the possibility of recall bias. However, some case-patients reviewed their credit card records for specific purchases. In addition, some case-patients required language translation. However this was not available for all case-patients, and some were hesitant to share information with investigators.

Additional limitations occurred during the food safety investigation in the identification of the unlicensed meat processor. While the meat processor was identified, the lack of contact with the meat processor severely impacted the progress of the investigation into identifying distribution channels of the implicated products. The limitations also included the lack of identification of the point in the supply chain where the contamination may have occurred.

The location and identity of the unlicensed meat processor of the shredded pork products are unknown. However, due to the higher proportion of shredded pork products in dishes served to the case-patients involved in the outbreak, local public health authorities were able to confirm the source of the outbreak through food sampling of the meals consumed by the cases. This action helped to ensure timely identification and removal of the implicated shredded pork product. Particularly in the absence of appropriate labels, photos of the unlabelled shredded pork products were pivotal in identifying the implicated products in the outbreak and for communicating information about affected food items to public health partners as well as to the hotels, restaurants and institutions.

## Conclusion

Outbreaks linked to food from unlicensed sources highlight the significant public health risk that arises from the use of these sources, such as the introduction of pathogens into the food supply chain and the propagation of disease ((22,23)).

These food products can be illegally distributed across a wide network, particularly at the restaurant level, thereby resulting in a greater risk for illnesses. The economic impact of rising food costs could influence the purchase choices of food safety operators in attempts to lower their operating costs, where affordable alternatives could be supplied from unregulated sources ((24)). Restaurant operators are also less likely to cooperate with investigators in providing information about products that they knowingly obtain from unlicensed sources. Given that a single type of cuisine was involved, this may also suggest a familiar network.

Prevention requires a multi-pronged approach, involving regulations that require food sources to be licensed and effective record-keeping. The Ontario Food Premises regulations were amended in 2019 to include these requirements. In addition, education of food safety operators on the risks to the public’s health of the use of foods from unlicensed sources, as well as the penalty that they would incur if they do so, may help reduce the risk. Furthermore, enforcement is an important approach to prevent such instances.

This outbreak highlighted the importance of collaboration among local, provincial and federal regulatory authorities (Ontario Outbreak Investigation Coordinating Committee). The use of additional risk-mitigating strategies, such as the inspection of all restaurants serving Southeast Asian food; the Ministry of Health issuing a notice to hotels, restaurants and institutions; and the Canadian Food Inspection Agency issuing a food advisory on the unlicensed shredded pork products, aided in identifying the product and removing it from the marketplace and increasing awareness of the implicated food product.
